# Treatment of Simple Ulcer of the Stomach

**Published:** 1871-05

**Authors:** H. Ziemssen

**Affiliations:** Professor of Clinical Medicine in the University of Erlangen, Bavaria


ART. II.—*Treatment of Simple Ulcer of the Stomach.* By H. ZIEMSSEN, Professor of Clinical Medicine in the University of Erlangen, Bavaria.

(From collection of Clinical Lectures in combination with German Clinical Professors, edited by Richard Volkman, Professor of Clinical Surgery in the University of Halle, Prussia, V. 15, 25th January, 1871.)

TRANSLATED BY GEO. NIEMEIER, M. D., FOR THE "BUFFALO MEDICAL AND SURGICAL JOURNAL."

(Continued From Last JOURNAL.)

Regarding the time of life there is not the same unanimity: the main point is to determine at what age the ulcer at first commences, not at what age it leads to death or is accidently found at dissections. Clinical experience teaches, that only a small fraction of patients sink under the first attack; that the majority attain middle or even old age, and perish by relapses, or by the sequels of the ulcer, and of other diseases; it chiefly attacks young and middle aged persons. I have to mention of those conditions causing the disposition in young individuals, changes of the chemical contents of the blood, and of the nutrition, in connection with the bodily development during and after puberty, chiefly chlorosis and anemia. We infer a priori from the frequency of the coincidence of these chemical changes with ulcer a casual connection; and Virchow, and Rokitansky, proved, in chlorotic females, the walls of vessels to be extremely thin, frequently with a premature fatty degeneration, and their lumen narrow, which, as a matter of course, exposes the smallest arteries and capillaries to be more easily torn, and makes it probable that relatively trifling injuries can lead to a circumscript hemorrhagic infiltration. The combination of ulcers with tuberculosis, and chronic pneumonia, is also frequent. I cannot prove, at present, certain casual connection of both; and we have to leave it undecided whether this combination is only merely accidental, caused by the absolute frequency of both processes; or, if not,rather the weakness of the constitution, and the various alterations in the nutrition of all tissues, consequently those of the walls of vessels likewise found in individuals with hereditary disposition to phthisis already at an early age—remember the disposition to bleeding from the nose, and haemoptysis—serve as a simultaneous issue of both processes. It is possible that the alteration of assimilation and general nutrition, as a consequence of the ulcer, hasten the dedevelopment of the lung affection. At least I have often observed that very obstinate ulcers preceded the latter for years. You will now understand that diseases of the endocardium, and of the tunica intima of vessels, are very frequently combined with ulcers, caused, as they are, through embolism and thrombosis of the minutest arterial branches of the stomach. How disturbances of innervation can lead to disturbances of circulation in the wall of the stomach is yet undecided; but the nerves of vessels play certainly an important part in diseased organs regarding circulation and nutrition. As occasional causes, I mention all those noxious substances to which the stomach is exposed, especially those which cause or maintain hyperaemia and inflammatory conditions in the mucous membrane, such as hard indigestible food; too hot or too cold, alcoholic, or otherwise, irritating liquids. My experience shows a frequency of the ulcer in professional cooks, easily to be explained in this manner. I might be induced to believed a traumatic cause of the circumscript hemorrhagia not quite so rare. Blows externally; compression of the stomach, caused by long and sitting posture; tight lacing in women; belts in men, especially during digestion; pressure to which the stomach is exposed on accountof the abdominal press during vomiting or hard evacuation of the bowels; all these mechanical actions may be able, provided the walls of vessels are tender, especially under a concurring digestive hyperaemia, or may be a catarrhal irritation, to produce rupture of small vessels or hemorrhagic infiltrations.

Now for the anatomical changes. The first changes are not yet sufficiently studied. The hemorrhagic infiltrated part, after the disturbance of nutrition, rapidly appears to scab and soften; we then find a sharp, limited, rounded part of the mucous membrane changed into a blackish pulpous or mory dry scab, the loosening of which shows the submucous or the muscular; or, after the destruction of both, the serous membrane. We have frequently a chance to study this early stage in extensive burns of the external skin, or if the ulcer was caused by mechanical violence or embolism. Rindfleisch found in a man who, on account of a strangulated hernia, vomited severely several days, and at last masses streaked with blood, besides several smaller hemorrhagic infractions, two circular spots in the curvatura minor, of which the one proved to be a hemorrhagic infraction of the mucous membrane, while the other showed all the characters of a pure ileus simplex. This observation makes it probable that necrosis, and the loosening of the necrotic mass, takes place rapidly under the influence of the gastric juice. The loss of substance ensuing after the loosening of the scab is of a longer duration, and therefore found more frequently. The extent of the primary defect, in breadth and depth, apparently depends principally upon the extent of the defective circulation; that is, upon the size of the impervious vessel or upon the extent of the hemorrhagic infiltration; but we always observe in penetrating, especially in perforating ulcers, the defect in its depth renewed terrace like. The further changes, the gradual increase of the loss of substance, must be ascribed principally to the peptic influence of the gastric juice. This deleterious influence of the gastric juice, or of the corroding products of the acid fermentation of the contents of the stomach, especially aretic and butyric acid, is explained partly by the loss of the epithelial coats, partly from the insufficient supply of alkalic blood to the ulcer. The alkalies of the blood do not sufficiently neutralize the acid penetrating the tissues, in consequence of the circulatory derangement, as Virchow pointed out at first. It depends upon the state of the vessels in the ulcer, and upon the qualities of the contents of the stomach, whether the ulcer extends rapidly or slowly; whether it is perforating or spontaneously, granulating or cicatrizing. It is evident that the blood-vessels must be corroded by progressive corrosion of tissues; but hemorrhages, especially severer ones, are comparatively rare, only one-third or one-fourth of all cases, because the erosion of smaller vessels is preceded by thrombosis of their lumen, and larger ones are only affected by a very deep penetrating destruction. The erosion of a larger artery must necessarily lead to a fatal hemorrhage, most frequently the A. gastroduodenalis, coronaria ventriculi, and gastro-epiploica dextra in the curvatura minor and pyloric portion. I observed an almost momentary fatal hemorrhage from the A. lienalis. Extensive destructions of the stomach-wall, in slowly progressing ulcers, are not nearly so destructive to life, as those acute and more latent ones: the latter frequently perforate the wall in its entire thickness, especially the anterior wall. In more slowly progressing ulcers of the curvatura minor, and neighborhood, the conglutination of the serous membrane with the surrounding parts, such as the pancreas, or the left lobus of the liver, puts a stop to the destruction; but these firm conglutinations of the stomach with the pancreas or liver, even after thorough healing of the ulcer, often lead to a permanent impediment of the free mobility, and of the peristaltic action of the stomach, and aided by the cicatricial contraction of the wall, cause contortions and changes in its form, impeding its digestive motions, and probably causing the severe cardialgia, which so frequently continue after the ulcer has thoroughly healed. An amelioration, even a cure, can spontaneously ensue, in spite of firm conglutinations, in the course of years, provided the bottom of the ulcer is yet formed by the stomach wall, and not by a neighboring organ. The original conglutination is gradually elongated by the peristaltic motions of the stomach, and changed into a string-like adhesion, which latter, by continuous pulling, gets thinner and finally ruptures. This natural cure may be noticed in cicatrices, and thickened spots of the serosa, without conglutination with the surrounding parts, the same as in partial adhesive pleuritis and pericarditis with the pleura and pericardium. The sequels of a cicatrix of large annular ulcers near the ostia, or curvatura minor, are more serious: the progressive cicatricial contraction causes strictures of the ostio, especially of the pylorus, or hour-glass strictures of the whole stomach, highly disturbing its functions. And this cicatricial stricture of the pylorus, with secondary gastrectasia, is often, many years after the healing of the ulcer, the subject of treatment.

The diagnosis of the ulcer is, in many cases, without difficulties; the coincidence of the more important symptoms, principally the cardialgia, disturbance of digestion, vomiting, hemorrhages, the long continuance of the complaint, with a relatively good condition of nutrition, and frequent pauses—youth or middle age make a certain diagnosis possible; but every experienced physician knows how frequent one or more of these more important symptoms are absent. The differential diagnosis, in middle aged people, between ulcer, cancer, and chronic catarrh, may be impossible, if the disease is not yet of long standing, without hemorrhages, no severe pain, if the physical exploration gives no positive result, and the nutrition of the whole body not lowered. The diagnosis is not less difficult in young individuals of a chlorotic and anaemic tendency, if the disturbances of nutrition are not yet of long duration, with trifling pain and sensibility of the epigastrium, if neuralgic pains exist also in other parts, and hemorrhages, cardialgia, and vomiting are absent after meals; the diagnosis frequently becomes more distinct in course of time through hemorrhage and perforation. But the diagnosis, with a favorable issue, remains doubtful even to the end of the treatment; and we may judge possibly in consequence of a relapse upon the nature of the former disease. These difficulties of the differential diagnosis of the ulcer, often quite paradoxical in its symptoms, from nervous cardialgia of chlorotic females, or of young women, suffering with uterine disease, from chronic catarrh and cancer in older people, make it desirable, in doubtful cases, to draw a conclusion from the success or nonsuccess of the therapeutical remedies upon the nature of the affection. The results of a careful treatment with alkalies may be cautiously used for the diagnosis, as a large majority of ulcers, and chronic catarrh, are either cured or improved for a longer period by it, while the sym toms of cancer and nervous cardialgia are either not changed at all or absolutely aggravated by it.

I come now to the most important part of my lecture, the treatment. All authors, since Cruveilhier, consider dietetic regime as most important, even many as the main point, and expected only a palliative effect from medicines, such as bismuth, argent nitr., plumb. acet. alum, tannin, ferri perchl. Oppolzer and Niemeier recommended highly the Carlsbad spas. Every physician agrees that narcotics are indicated against cardialgia, ice and cold against hemorrhage, carbonate of soda against the acidity of the stomach. Let us put three questions: 1. What conditions are favorable to the formation of the ulcer? 2. What movements cause the enlargement and dangers of it? 3. What, at last, are the impediments of its spontaneous cure? I may answer to the first question, that we may often prevent the ulcer provided that we are able to remove those casual conditions by early treatment of chlorosis and anemia, regulation of the diet, &c., &c. These are the points for a rational prophylasis of the ulcer. The two other questions are, practically, of more importance, for we are only rarely in a position to use these prophylactic measures; far more frequently we see the ulcer long after its formation, neglected and maltreated, and my answer is: The first and most important object is the neutralization of the acidity, in whatever stage you see the ulcer, in order to protect the young granulation from the influence of the acid and pepsin. For such a neutralization, a simple watery solution of Carb. of Soda, little concentrated, and in larger quantities in an empty or only partially filled stomach would be sufficient, provided this could be done continually, and this would make the assimilation of albuminous substances impossible, and gravely injure the general nutrition. Such a continuous neutralization is not necessary at all; once a day is ufficient, under the condition that at least once a day the sour contents of the stomach are completely emptied into the bowels, and for this purpose I recommend the sulphate of soda, which powerfully excites the peristaltic motion of the stomach witnout irritating the mucous membrane and the ulcer, it promptly causes the evacuation of the stomach, and to a certain extent reduces or even prevents the sour fermentation of its contents. Common salt—Chloride of soda–acts similarly, but far weaker as an anti-fermentative and antiseptic, and slightly stimulates the muscular membrane of the stomach and intestines; but this effect, is, in most individuals, quite insufficient. These three substances, sulph. of sod., carb. of sod., and chloride of sodium, are the principal constituents of the celebrated Carlsbad Spring Waters. I consider the permanent intensely sour quality of the stomach, caused, not by the ulcer, but by chronic Catarrh, as especially injurious for the decrease of the [illegible] Dyspepsia, eructations and vomiting, pyrosis, the permanent sensibility and tympanitis in the epigastrium, the costiveness, emaciation and general anemia, are mostly caused by chronic catarrh. The mucous, produced in abnormal quantities, acting as ferment, causes a sour fermentation so much quicker, the less carbonhydrate the food contains—people in general, have an aversion against meat and other food containing proteine, and such food is the more disposed to fermentation the longer it remains in the stomach, and this latter condition, the obstinate delay of the chyme in the stomach deserves especial attention. It is a priori very probable, that this delay and the vomiting may be caused by a temporary mechanical obstruction or stenosis of the pylorus; still we often find after death, the pylorus sufficiently pervious; in other cases the frequent changes of the symptoms, the temporary complete disappearance of all symptoms leading to a suspicion of a stricture of the pylorus, but mainly the thorough recovery of the patient, proves that there was only a temporary obstruction of the pylorus. We are therefore to conclude that this mechanical obstruction is caused by the catarrhal swelling of the mucous membrane of the pylorus, together with a spastic contraction of its sphincter,that the energy of the muscularity of the stomach decreases the longer the ulcer and catarrh continue, and that the contractions are not more than sufficient to open the closed sphincter. Kussmaul lately proved. by experiments with the stomach pump,that the sour fermenting contents of the stomach, in spite of frequent and copious vomiting, are never entirely emptied. If there exists a certain degree of paresis of the muscularis and of gastrectasia, this remaining small portion of fermenting chyme produces at once upon all fermentative substances carried into the stomach the same change, so that this power of fermentation and the catarrh become permanent, and the ulcer is always under the corroding influence of sour chyme. The majority of patients are for a long time in this state before they seek medical advice. ***

You may give either the natural or artificial Carlsbad Spring Water, or the natural or artificial Carlsbad Salt in a more or less concentrated hot watery solution, to which latter I give the prefer-

*** It has become necessary on account of the length of the original article to abbreviate a little.

ence; the natural Carlsbad salt contains about 87 per cent. sulp. sod., about 13 per cent., carb. sod., and only a small quantity chlor. sod., (common salt.) †

A few words about the diet: Your patients should avoid most of the vegetables, fruit, fat, sugar, beer; you will allow them roasted veal and chicken, beef tea, raw ham, wheaten bread, light French Claret, milk in any quantity, either alone or to prevent rapid fermentation, with the addition of a little carb. sod., buttermilk. I generally give, for about four weeks, in the morning, fasting, two large teaspoons of either the natural or artificial Carlsbad salt, dissolved in a quart of water, previously boiled and moderately cooled, and have the fourth part of this solution taken every quarter of an hour, so that one or two watery evacuations follow; during the drinking, or shortly after it, I increase, if necessary, and in course of time decrease the dose of salt, giving at last only one teaspoonful in the same quantity of water. In severe cardialgia, I give morphine, either subcutaneously or by mixing 2 grains with half an ounce of Aq. amygd. amar., and giving of this repeatedly ten to fifteen drops. I have never seen any benefit of nitrate of silver, and only very temporary, but no curative results fron nitr. of Bismuth with opiates.

Let me say a few words about the treatment of hemorrhage and perforation: Hemorrhage, if it can be stopped at all, is treated the simplest and surest with cold—ice—externally and internally, perfect rest, and absolute abstinence from solid food and liquids; I don't advise you to use astringents at first, such as alum, perchl, iron, plumb., acet., they can never reach the bleeding vessels of the stomach if it is filled with coagulated blood, often increase the vomiting and so are apt, by loosening the provisional thrombus in the contracted lumen of the vessel, to renew the bleeding. On the 2d or 3d day I allow iced alumnwhey, in small quantities, an injection if necessary, but never a physic, not even the mildest; milk, beef tea, wheaten bread, iced champagne. Perforation, especially for the purpose of euthanasia, and to quiet the movements of the stomach and intestines so as to check the *extravasation* of the contents of the stomach into the peritoneal cavity—almost always only

† The translator has used for some twenty years an artificial Carlsbad salt, by mixing extemp, 1 ounce of sulph. sod., with 1½ teaspoonful of carb. sod., and 3-4 of a teaspoonful of common salt.

a pious wish—requires opium in large doses subcutaneously or per anum. I use ice in bladders against peritonitis and tympanitis.

I wish yet to draw your attention to the treatment of some consecutive troubles which require our care not seldom after the healing of the ulcer.

1st. To strictures of the ostia, caused by gradual cicatricial contraction near the pylorus or cardia. The cicatricial strictures of the ostia is of the greatest importance, and as we have seen the ulcers frequently near the pylorus, so we observe these secondary strictures mostly near the pylorus. We may safely put the diagnosis of cicatricial pylorus stricture, if the catarrh does not entirely stop or return with a retention and sour fermentation of the contents of the stomach, with vomiting, at first only at times, gradually more frequent, and at last quite regular after meals, obstinate constipation of the bowels, with a sinking of the hypogastric re-region, and a physical demonstration of gastrictasia. Less characteristic are the symptoms of a distortion or stricture of the central part of the stomach; they, also, are accompanied by disturbances of digestion and assimilation, cardialgia and vomiting. Strictures near the cardia are as seldom, as ulcers near the cardia are rare.

In the treatment of stricture near the cardia, I recommend a mechanical dilatation by a daily introduction of bougies, with gradually increasing tnickness, as used in strictures of the œsophagus from burns or caustics. In strictures of the pyloros, not being accsssible to treatment by bougies, if all the outer treatment fails, we may employ the stomach pump every morning; empty the stomach completely, and inject through it either natural or artificial soda water, in order to relieve the stomach, often enormously distended by its sour contents and gas.

2d. To the mechanical restriction of the peristaltic motion caused by conglutination of the stomach with neighboring organs. To a tonic dyspepsia and pyrosis, I order, in such cases, natural or artificial soda sulphates, containing small quantities of iron, or amara combined with a little iron, always using Rhubarb as a physic, if needed, but iron has to be cautiously used, and obstruction of the bowels carefully guarded against.

3d. To habitual constipation of the bowels, which generally folows the ulcer for months, even for years, I give Rhubarb, either as simple or comp. extr., regularly every night before going to bed, and combine with in extr. Bellad., or extr. Nux. Vom. spirit., until the stomach has regained its normal energy. A certain vulnerability of the mucous membrane of the stomach, however, will mostly remain, which forces patients to keep always a scrupulous caution in their diet, the stomach will always be a "*locus minoris resistentiae.*"

